# Design and Performance of a Portable and Multichannel SPR Device

**DOI:** 10.3390/s17061435

**Published:** 2017-06-19

**Authors:** Xiao-ling Zhang, Yan Liu, Ting Fan, Ning Hu, Zhong Yang, Xi Chen, Zhen-yu Wang, Jun Yang

**Affiliations:** 1 Key Laboratory of Biorheological Science and Technology, Chongqing University, Ministry of Education, Bioengineering College, Chongqing University, Chongqing 400030, China; zhangxiaoling@cqu.edu.cn (X.Z.); 20126461@cqu.edu.cn (T.F.); 20121913019t@cqu.edu.cn (X.C.); 2 Chongqing Engineering Research Center of Medical Electronics Technology, Chongqing University, Bioengineering College, Chongqing University, Chongqing 400030, China; 20141902049@cqu.edu.cn; 3 Department of Laboratory Medicine, Southwest Hospital, Third Military Medical University, Chongqing 400038, China; zyang@tmmu.edu.cn; 4 Department of Biomedical Engineering, Chongqing Medical University, Chongqing 400016, China; wangzhenyu20090306@gmail.com

**Keywords:** surface plasmon resonance, sensor, portability, microfluidic

## Abstract

A portable multichannel surface plasmon resonance (SPR) biosensor device is presented in this study. As an optical biosensor device, the core component of its light path is a semi-cylindrical prism, which is used as the coupling unit for the excitation of the SPR phenomena. Based on this prism, a wedge-shaped incident light beam including a continuous angle range (10°) is chosen to replace the commonly-used parallel light beam in traditional SPR devices, in which the incident angle is adjusted by a sophisticated mechanical system. Thus, complicated, cumbersome, and costly mechanical structures can be avoided in this design. Furthermore, the selection of a small and high-stability semiconductor laser and matrix CCD detector as well as a microfluidic system aids in the realization of a miniaturized and multichannel device. Several different samples were used to test the performance of this new device. For ethanol with different concentrations, the sensing response was of good linear relativity with the concentration (*Y* = 3.17143*X* + 2.81518, *R*^2^ = 0.97661). Mouse IgG and goat anti-mouse IgG were used as biological samples for immunological analysis, and BSA as the control group. Good specific recognition between mouse IgG and goat anti-mouse IgG has been achieved. The detection limit of antibody to antigen coated on the sensing surface was about 25 mg/L without surface modification.

## 1. Introduction

Surface plasmon resonance (SPR) biosensors are a widely-used optical sensor, capable of rapidly monitoring the interaction between two kinds of biomolecules in real-time, with no requirement for a label. Since Liedeberg and Nylander [1] set up a gas sensor based on the SPR phenomenon in 1983, SPR technology has been applied not only in gas detection [2], but also in biomacromolecular interaction (e.g., protein and protein [3,4], DNA and DNA [5], aptamer and ligand [6]) analysis, food safety [7], environmental monitoring [8,9,10], drug screening [11], and so on. Owing to its outstanding advantages compared with some other traditional methods such as enzyme-linked immune sorbent assay (ELISA) and radioimmunoassay (RIA), SPR biosensors have high value and huge potential use in biomedical analysis. However, some bottlenecks, such as cumbersome and complicated structure as well as high cost, must be overcome.

The research and development of miniaturized and high-throughput SPR sensors are attracting wide attention. For example, an SPR sensing device (Spreeta SPR2000) developed by Texas Instruments (USA) integrated a delicate optical structure in a miniaturized system [12]. Compared with traditional SPR biosensors with single or dual channels, multichannel design cannot only exclude the temperature fluctuation, non-specific binding, and background noise, but also detect more samples simultaneously to achieve high efficiency [13,14]. Increasing efforts have been made to realize the miniaturization and multichannel detection of SPR devices [11].

In this study, a miniaturized and portable SPR biosensor was developed. To realize the aim of miniaturization and portability, the complicated turntable structure which is used in traditional devices to achieve variable incident angles was replaced with a wedge-shaped incident light beam formed by a focusing lens. Meanwhile, a focusing lens with small size and short focal distance was adopted. Additionally, a small and high-stability semiconductor laser and matrix CCD detector were chosen. Aiming at multichannel monitoring and low sample consumption, a few micro-channels were fabricated and integrated with the sensing surface to realize the detection of multiple samples simultaneously. Finally, several samples were used to calibrate and test this new device.

## 2. Experimental

### 2.1. SPR Device

**Figure 1:**
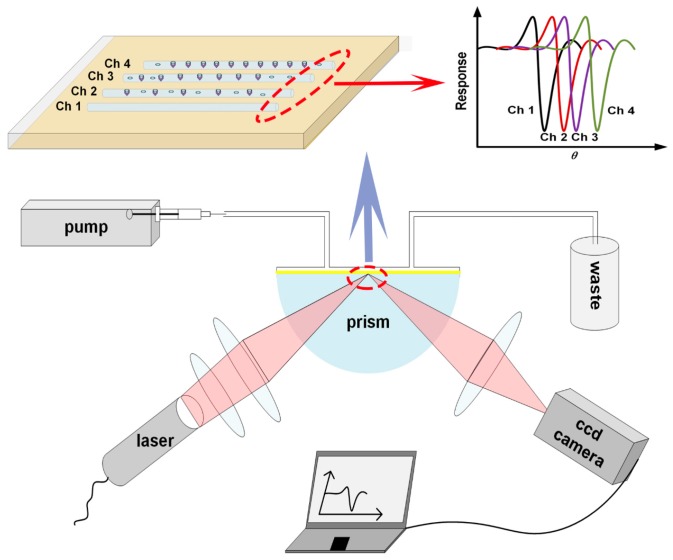
Sketch map of the optical system of the surface plasmon resonance (SPR) sensor device (Channel: CH).

An SPR biosensor is an optical sensor device, in which the optical path is the most important unit. This device is based on an angle-modulated mode, and the reflectivity of different incident angles is detected to achieve the SPR angle (the angle of the lowest reflectivity). In the optical path design (Figure 1), the core component is a semi-cylindrical prism. A parallel light beam from the source will be converged to form a wedge-shaped incident light beam, which will be focused on the bottom of the prism. A sensor chip with a 50 nm-thick gold film is bound on the bottom of the prism, and the SPR phenomenon on the gold film will change the reflectivity of incident light. Biological reaction may influence the SPR phenomenon, and hence change the SPR angle. The wedge-shaped incident light beam includes a series of continuous incident angles, so the reflectivity of these angles can be monitored by using a photoelectric detector, and complicated mechanical rotation structures can be omitted. This optical system is integrated with a microfluidic system as well as a controlling and processing module to form the sensor device (Figure 1). 

The thickness of the protein film *l* on the surface of the gold film is constant, and the refractive index of the protein film on the surface of the gold film increases with the adsorption of protein molecules. Assuming that the protein concentration of the layer of protein film on the surface of the gold film is *C* after a period of time *t*, the surface concentration of the protein on the surface of the gold film is *Γ* = *Cl*. With the adsorption of protein molecules on the surface of the gold film, the change of protein concentration in the membrane leads to the change of the surface concentration, which also increases the refractive index of the protein membrane:

(1) $$\Delta \Gamma =\Delta C\cdot l=\frac{\partial C}{\partial n}\Delta n\cdot l=\frac{\partial C}{\partial n}\Delta (nl)$$

Using the linear relationship between the effective optical path difference and the resonance angle, the relationship between the SPR pixel shift and the protein surface concentration adsorbed on the surface of the gold film was obtained as follows [15]:

(2) $$\Delta P=A\frac{\partial n}{\partial c}\Delta \Gamma$$

ΔP is pixel shift, and *A* is constant. When the light source chosen is red light with a wavelength of about 650 nm, ∂*n*/∂*c* ≈ 0.18 [16], the relationship between SPR pixel changes and the biofilm concentration in the gold film surface is:

(3) ΔP=0.18AΔΓ

#### 2.1.1. Optical System

A small semiconductor laser (77 L × 30 W × 30 H mm^3^, 0.2 kg, PRL-FS-637, New Industries Optoelectronics, Changchun, China) was chosen as the light source. A monochromatic laser beam (637 nm, 0.1 mW) of 3 mm diameter passes through a polarizer to form *p*-polarized light. It is converged by using a plano-convex cylinder lens to form a wedge-shaped light beam. By choosing a shading plate with a suitable rectangular orifice, the angle range of the wedge-shaped light beam can be adjusted to 10°. This light beam enters the K9 glass semi-cylindrical prism from one side of the cylindrical surface and is focused on a line at the bottom plane. The reflective light beam, which passes through another side of the cylindrical surface of the prism, is detected by using a CCD camera (Seek, CSK-802B, GlenSeek Electronics, Shenzhen, China). The sensing area of this monochrome CCD camera is 582 pixels (W) × 512 pixels (H), and the distance between two neighboring pixels is 8.3 μm, which corresponds to a 0.015° variation of the reflective angle in our devices.

#### 2.1.2. Microfluidic System

**Figure 2:**
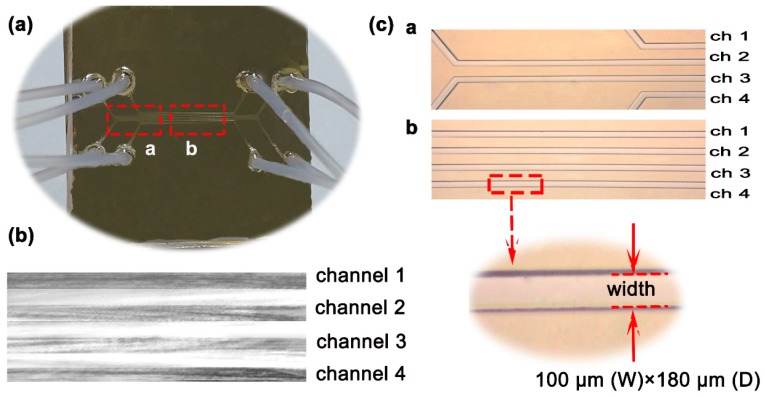
Sensor chip with integrated microchannels. (**a**) A polydimethylsiloxane (PDMS) slab with microchannels was bonded on the gold surface to form a closed microfluidic network (Channel: CH); (**b**) CCD image of the reflective light beam from the sensor chip; (**c**) Microscopic image of microchannels (a. the end of microchannel, b. the middle of microchannel).

The microfluidic system is another important part in this SPR device for sample control. There are four micro-channels flowing through the sensing surface, so four reactions can be monitored simultaneously. The microfluidic network is fabricated on a polydimethylsiloxane (PDMS) slab, and each microchannel is 1000 μm (L) × 100 μm (W) × 180 μm (D). The fabrication of the PDMS chip was based on a traditional replica molding method, in which the master was fabricated by using SU 8 photoresist on a silicon wafer by the photography method [17]. Input and output tubes were bonded on the microfluidic chip. After the oxygen plasma treatment of the gold surface and the PDMS slab, the PDMS slab was covered on the gold surface to form a closed microfluidic network (Figure 2).

### 2.2. Materials

Ethanol was purchased from Dongfang Chemical (Chongqing, China). Phosphate-buffered saline (PBS, pH 7.4), bovine serum albumin (BSA), mouse immune globulin G (IgG) and goat anti-mouse immune globulin G (anti-IgG) were purchased from Jianglai Biological (Shanghai, China). Deionized water was used for the preparation of all solutions.

## 3. Results and Discussion

### 3.1. Ethanol Calibration

**Figure 3:**
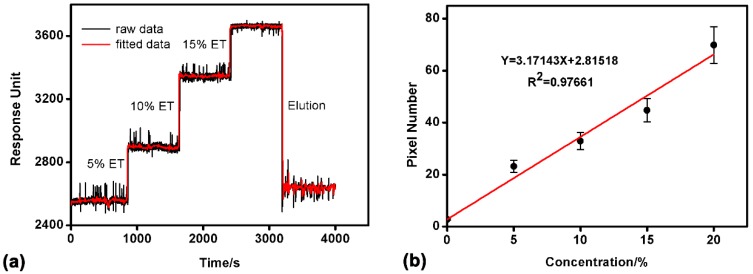
Ethanol detection in the SPR device. (**a**) SPR response of variable ethanol concentrations from 5% to 15% (Ethanol: ET); After washing, the SPR response curve almost returned to the baseline; (**b**) the calibration curve of variable ethanol concentrations. The fitting relationship is *Y* = 3.17143*X* + 2.81518, the goodness of fit *R*^2^ = 0.97661 (repeat times n = 10).

For calibration of the sensor device, ethanol solutions with concentration 5–20% were detected. Before sample loading, deionized water was loaded to rinse the microfluidic network and then the detection baseline was achieved. In the detection of ethanol solutions, the flow rate of each sample was 50 μL/min. For this SPR device, the change of SPR angle corresponds to the location shift of the pixel with the lowest reflectivity. Thus, the SPR response of each ethanol solution could be represented as the location shift of the pixel (with the lowest reflectivity) from that of the deionized water (baseline). For each concentration, the test was repeated 10 times. The experimental results (Figure 3) showed good linear relationship (*Y* = 3.17143*X* + 2.81518, *R*^2^ = 0.97661, n = 10) between the SPR response and the concentration of ethanol. Response unit refers to CCD camera pixels, and pixel number means CCD camera pixels increment.

### 3.2. BSA Adsorption

**Figure 4:**
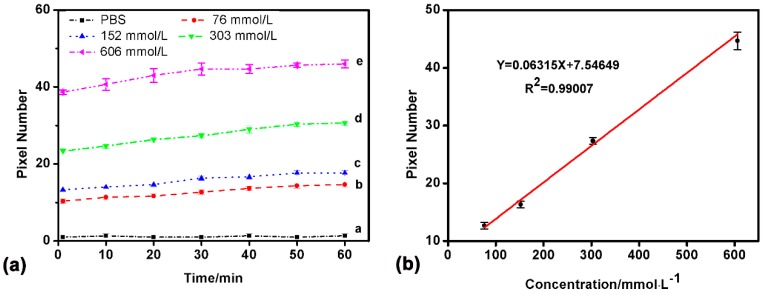
SPR detection results of the bovine serum albumin (BSA) adsorption. (**a**) The difference of the SPR response of 30 min was just about 5% less than that of 60 min, and 20% larger than that just loading the sample; (**b**) The linear fitting relationship of BSA of different concentrations (76–606 mmol/L), *R*^2^ = 0.99007 (repeat times n = 3). PBS: phosphate-buffered saline.

BSA (molecular weight ~66 kDa) is commonly used to block out the redundant binding sites on the sensor chip, and was used as a protein sample to test the on-chip protein adsorption. In this experiment, PBS solution was used to prepare BSA solution (76–606 mmol/L) and as the running buffer. For each sample, the flow rate was 50 μL/min. For BSA with different concentrations, the detected results showed that the adsorption was basically stable after 30 min. The adsorption of four different samples could be monitored simultaneously. One channel was used as a reference channel to eliminate the background noise including temperature fluctuation, non-specific adsorption, and so on (Figure 4). The difference of the SPR response of 30 min was just about 5% less than that of 60 min, and 20% larger than that just loading the sample. This means that the full adsorption of BSA on the sensing surface required nearly 30 min. For BSA adsorption, the fitted curve of the detection results was also of good linearity (*R*^2^ = 0.99007), which indicates that there is a good liner relationship between pixel numbers and BSA concentration for this home-made sensor.

### 3.3. IgG Immunological Reaction

IgG is the main ingredient of the serum immunoglobulin (up to 75% of total). It plays a very important role in the immune response, and hence is regularly used in immunological analysis. In this SPR device, analyte was coated on the sensor chip by physical adsorption [18] based on the electrostatic and hydrophobic interaction, so that the disturbance of chemical modification could be avoided. If IgG was adsorbed on the sensing surface, the physical adsorption may hide some binding sites on the “Y”-shape [19,20] structure and disturb the interaction between antigen and antibody. However, free antibody in the solution may expose sufficient bonding sites, and antigen is immobilized on the chip here.

Before the immunological analysis experiment, the sensor chip was immersed and washed in ethanol, followed by being dipped into DI water and dried by nitrogen. Both mouse IgG, BSA, and goat anti-mouse IgG were dissolved in PBS solution. In order to guarantee that the mouse IgG could cover most of the gold surface, a high concentration (1 g/L) was adopted to decrease the nonspecific adsorption between the sensing surface and antibody. After the antigen adsorbed on the surface for 30 min followed by PBS flowing to remove loosely bound molecules, BSA solution of high concentration (152 mmol/L) was loaded to occupy blank sites on the surface. Antibodies with different concentrations (25–500 mg/L) were loaded.

**Figure 5:**
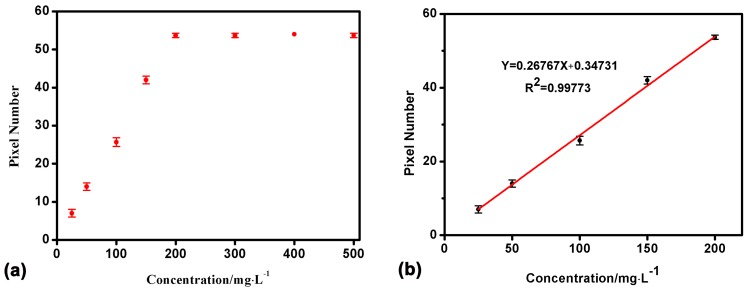
Detection results of the IgG immunological reaction. (**a**) Shift of the pixel increment for different concentrations of anti-IgG; (**b**) Below the saturation concentration (25–200 mg/L), the interrelationship between the concentration and response is linear *R*^2^ = 0.99773 (repeat times n = 3).

The IgG immunological reaction result is shown in Figure 5. As the concentration of anti-IgG increases between 25 mg/L and 200 mg/L, the sensing response increases gradually (Figure 5a). However, when the concentration exceeds 200 mg/L (e.g., 200–500 mg/L), the response has little increase. The saturation of the response signal may be induced by the limited binding sites of the coated IgG on the surface. When BSA is used to block the blank sites on the sensing surface, the infinitesimal SPR response (shift of the pixel) indicates that the concentration of 1 g/L IgG is high enough to coat most of the sensing surface. Below the saturation concentration (25–200 mg/L), the interrelationship between concentration and response is linear (*R*^2^ = 0.99773, Figure 5b).

In this device, BSA was used as a control group in order to verify the specific binding between mouse IgG and goat anti-Mouse IgG. BSA with the same concentration as IgG was coated on the sensing surface at first. Experimental results (not shown here) indicated that little response could be detected when goat anti-IgG was loaded on the BSA-coated surface.

## 4. Conclusions

In this paper, we aimed to develop a lightweight and multi-channel SPR sensor device. A semi-cylindrical prism was used as a coupling unit for the SPR excitation. The adoption of a wedge-shaped incident light beam could avoid the use of a complicated, cumbersome, and costly mechanical structure. Some other carefully-chosen components such as a small semiconductor laser light source with good light stability helped the realization of the portability of this device. In addition, a microfluidic chip was used as the core of the sample manipulation system for more channels and low consumption. Several experiments were carried out in this new device, and good precision and linearity of the response could be achieved. The home-made multichannel SPR sensors had a good performance in detecting the biomolecule interactions.
